# Long-term clinical and biochemical residue after COVID-19 recovery

**DOI:** 10.1186/s43066-021-00144-1

**Published:** 2021-09-12

**Authors:** Mohammed Ali Gameil, Rehab Elsayed Marzouk, Ahmed Hassan Elsebaie, Salah Eldeen Rozaik

**Affiliations:** 1 Endocrinology Unit, Internal Medicine Department, Faculty of Medicine, Mansoura University, Mansoura, Dakahlia Egypt; 2 Medical Biochemistry Department, Faculty of Medicine, Mansoura University, Mansoura, Dakahlia Egypt; 3 Clinical Pathology Department, Faculty of Medicine, Mansoura University, Mansoura, Dakahlia Egypt; 4 Gastroenterology and Hepatology Unit, Internal Medicine Department, Faculty of Medicine, Mansoura University, Mansoura, Dakahlia Egypt

**Keywords:** Post-COVID-19, Systemic changes, Long-term consequences

## Abstract

**Background:**

The long-term health consequences of coronavirus disease 2019 (COVID-19) are still unclear. The majority of previous trials addressed the post-COVID-19 symptoms through comprehensive medical questionnaires for relatively short periods after recovery. We tried to detect the potential pathological clinical signs and biochemical residue which persist for more than 3 months after the negative real-time polymerase chain reaction (RT-PCR) test of SARS-CoV-2.

**Results:**

Among 120 COVID-19 survivors of mean age 38.29 and 55.6% male proportion, systolic blood pressure was significantly elevated (***P***=0.001). Erythrocyte sedimentation rate (ESR), C-reactive protein (CRP), D-dimer showed higher values in COVID-19 survivors (***P***< 0.001). Alanine aminotransferase (ALT), aspartate aminotransferase (AST), gamma-glutamyl trans-peptidase (GGT), and alkaline phosphatase (ALP) were significantly elevated in contrast to serum albumin that was reduced in COVID-19 survivors (***P*** ≤0.001). Serum lipase, amylase and albuminuria were higher in COVID-19 survivors (***P*** ≤0.001). Regression analysis (AOR, 95% CI) showed that ESR (***P*** = 0.014), haemoglobin concentration (***P*** = 0.039), serum lipase (***P***= 0.018), blood urea nitrogen (***P***= 0.003), albuminuria (***P***= 0.046), 25(OH) vitamin D (***P***= 0.002), and serum uric acid (***P***= 0.005) were the significant predictors of COVID-19 survivors (94.8% an overall prediction).

**Conclusion:**

COVID-19 survivors experienced residual significant clinical and biochemical alterations that necessitate comprehensive medical care and close follow-up for longer periods.

## Background

At the end of 2019, China declared an epidemic of a novel coronavirus, systemic acute respiratory syndrome coronavirus 2 (SARS-CoV-2) which spread globally causing the coronavirus disease 2019 (COVID-19). The World Health Organization (WHO) considered the COVID-19 as a pandemic on 11th March 2020 [1]. SARS-CoV-2 is the most recently evolved coronavirus which is an enveloped single-stranded RNA virus. This novel virus shares high similarity to severe acute respiratory syndrome coronavirus (SARS-CoV) that caused an epidemic, in 2003 [2]. The world is still struggling against COVID-19. Block spread of this potentially lethal virus is the highest priority for all nations. COVID-19 is the most serious threat to public health as no specific curable therapy is approved till no w[3]. Angiotensin-converting enzyme 2 (ACE2) is a common host cell receptor for SARS-CoV and SARS-CoV-2. ACE2 is diffusely dispensed throughout human tissues such as in lungs, kidneys, small intestine, testicles, myocardium, thyroid gland, pancreas, adipose tissues, blood vessels, blood cells, spleen, bone marrow, brain, liver, urinary bladder, and adrenal glands [4]. COVID-19 has exhibited a wide variety of clinical manifestations with a diverse prognosis. The majority of patients had reversible systemic manifestations such as fever, sever bone pain, dyspnoea, cough, and diarrhoea but the minority may experience a critical illness, multisystem organ failure with disastrous burden on the health care system [5].

Post-COVID-19 manifestations after negative real-time reverse transcriptase-polymerase chain reaction (RT-PCR) test of SARS-CoV-2 had been largely studied in the literature. Kamal et al. [6] found a large similarity between the post-COVID-19 symptoms and the post-SARS-CoV. Meanwhile, Dana et al. [7] doubted the implication of long-term health consequences of severe acute respiratory syndrome coronavirus (SARS-CoV) and Middle East respiratory syndrome coronavirus (MERS-CoV) on patients recovered from COVID-19. The majority of previous trials addressed the post-COVID-19 symptoms through comprehensive medical questionnaires for relatively short periods after recovery. They proposed the link between COVID-19 severity and post-discharge manifestations through epidemiological studies. A Facebook survey for the post-COVID-19 symptoms was conducted by Yvonne et al. [8] who reported persistence of symptoms in the majority of COVID-19 survivors. Also, Carfì et al. [9] conducted a post-acute COVID-19 comprehensive questionnaire to detect the persistent symptoms after COVID-19 recovery in the follow-up period (14–60 days). They reported persistent at least one COVID-19-related symptom in the majority of patients.

However, to our best knowledge, little is known about the pathological residue which may underlie the post-COVID-19 manifestations that necessitate comprehensive medical follow-up care. We tried to detect the significant clinical signs and biochemical alterations in COVID-19 survivors for relatively long periods after recovery.

## Methods

A case-control study was conducted at the outpatient department of Mansoura University hospital during the period from August 2020 to December 2020, included 240 participants divided into two groups of matched age and sex. Case group comprised of 120 participants with prior COVID-19 that was diagnosed with RT-PCR test followed by proven negative RT-PCR test of SARS-CoV-2 for at least three months before enrolment. All participants of the case group experienced an irrelevant medical history either before COVID-19 or throughout post-recovery period till enrolment. Participants with a history of mild to moderate COVID-19 illness without admission or oxygen therapy were selected to avoid confounding influence of severe illness. The control group included 120 healthy participants without history of COVID-19 or other morbidities. Hereby, we intended to compare COVID-19 survivors who are expected to resume their full wellness with non-COVID-19 exposed subjects. COVID-19 survivors were recruited by phone call for follow-up and face to face interview at pulmonology clinic after more than 3 months period of documented negative RT-PCR test of SARS-CoV-2. Out of 168 COVID-19 survivors, only 120 agreed for participation. Participants were subjected to detailed medical history taking, clinical examination with anthropometric measures and laboratory assessment of fasting lipid profile, serum lipase, amylase, serum creatinine, estimated glomerular filtration rate (eGFR), blood urea nitrogen (BUN), morning spot urine albumin creatinine ratio (urine ACR), Alanine aminotransferase (ALT), aspartate aminotransferase (AST), gamma-glutamyl trans-peptidase (GGT), alkaline phosphatase (ALP), serum total bilirubin, serum albumin, complete blood count (CBC), erythrocyte sedimentation rate( ESR), C-reactive protein( CRP), ferritin, D-dimer, serum uric acid, and 25 (OH) vitamin D with abdominal and pelvic ultrasonography.

### Exclusion criteria

We excluded patients with pre-existing; before COVID-19, acute or chronic morbidities such as diabetes mellitus, endocrine disorders, autoimmune diseases, acute or chronic infections, cardiac, renal, hepatic impairment, primary biliary disorders, gall bladder disease, choledocholithiasis, alcohol consumption, or current use of medicine that may alter participants’ findings like steroid, hormonal therapy, anabolic steroid, non-steroid anti-inflammatory drugs ( NSAIDs), diuretics, chemotherapy, or biological therapy. We excluded patients with a history of severe COVID-19, hospital admission, or oxygen therapy. We excluded COVID-19 survivors who developed acute or chronic illness during the period from negative RT-PCR test of SARS-CoV-2 till enrolment time for the possibility of perturbation of body parameters.

### Ethical approval

The official approval was obtained from the Institutional Review Board (IRB) for the Clinical Research Committee of Mansoura University with approval number (No.R.20.06.1158.R1). All procedures performed were in accordance with the ethical standards of the institutional research committee and with the 1975 Helsinki Declaration. Written consent for participation was approved by the IRB and obtained from all participants.

### Statistical analysis

Data were processed with IBM. SPSS Statistics for Windows, Version 22.0. Armonk, NY: IBM Corp. number and percentage described the Qualitative data meanwhile the Quantitative data were represented as median and interquartile range for non-parametric data and mean ± standard deviation for parametric data after Kolmogorov-Smirnov test of normality. Significant values were judged at the (0.05) level. Student’s *t* test and Mann-Whitney *U* test were used for bi-variable analysis. Binary logistic regression analysis was applied for all studied variables. The significant predictors were processed via forward Wald method/enter regression model with adjusted odds ratios and 95% confidence interval.

## Results

This study included 120 COVID-19 survivors with documented negative RT-PCR test of SARS-CoV-2 with mean age 38.29±5.27 compared to 120 healthy participants without a history of COVID 19 with mean age 37.25±4.87. Gender was matched among participants. Male participants represent 55.8% 55.6% and 57.5% 58.7% of case and control groups, respectively with a non-significant difference (*P*=0.695) as shown in Table 1. Systolic blood pressure was significantly elevated in COVID-19 survivors (*P* =0.001) (see Table 1).

**Table 1:** The clinical characteristics of participants

Variable	Control group *N* = 120	Cases group *N* = 120		Test of significance	*P* value
	Mean ± SD/No (%)				
Age (years)	37.25±4.87	38.29±5.27		t=1.23	p=0.22
Sex: No (%)					
Male Female	69(57.5) 51(42.5)	67(55.8) 53(44.1)		χ^2^=0.152	p=0.695
Participants No (%) According to Period after negative RT-PCR test	-	3-4 months	58(48.3)	-	-
	-	4 -5 months	37(30.8)		-
	- -	5-6 months	15(12.5)	-	
		> 6 months	10(8.3)	-	-
Body weight (kg)	80.21±9.38	82.96±8.78		t=1.85	p=0.07
Height (cm)	170.48±6.26	170.48±6.94		t=0.001	p=0.999
BMI (kg/m^2^)	27.77±4.31	28.76±4.42		t=1.38	p=0.170
SBP (mmHg)	120.63±8.49	126.70±10.31		t=3.84	p=0.001*
DBP (mmHg)	77.86±7.05	79.94±7.32		t=1.76	p=0.08

*t*: Student’s *t* test *statistically significant (if *p* < 0.05)

**Fig. 1:**
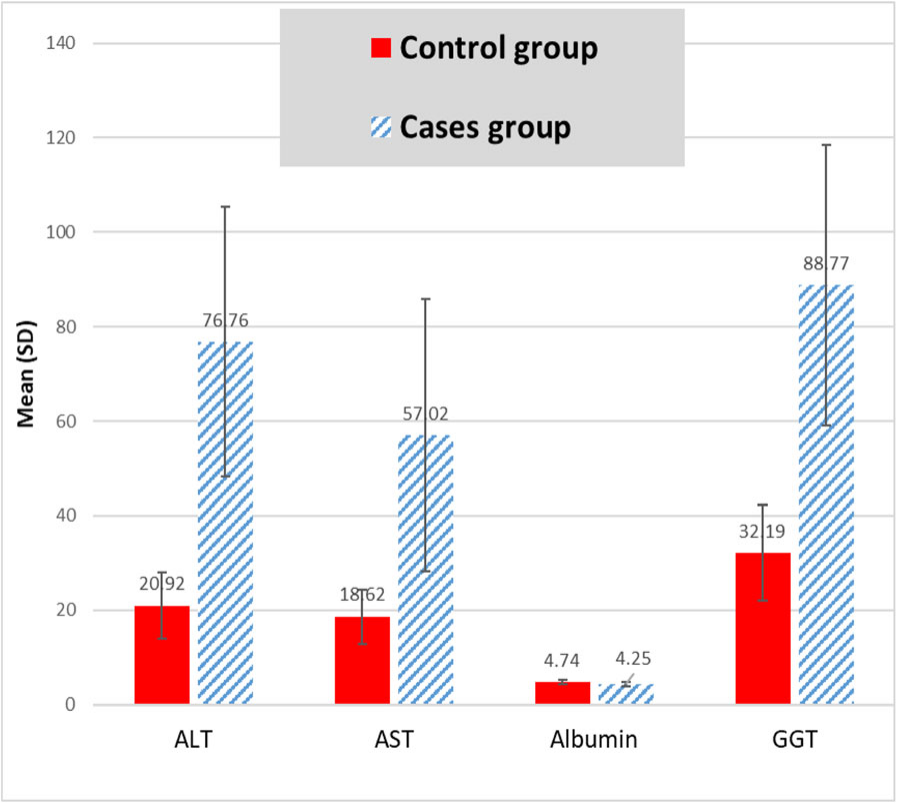
Significant differences in the liver function tests between the studied groups

**Fig. 2:**
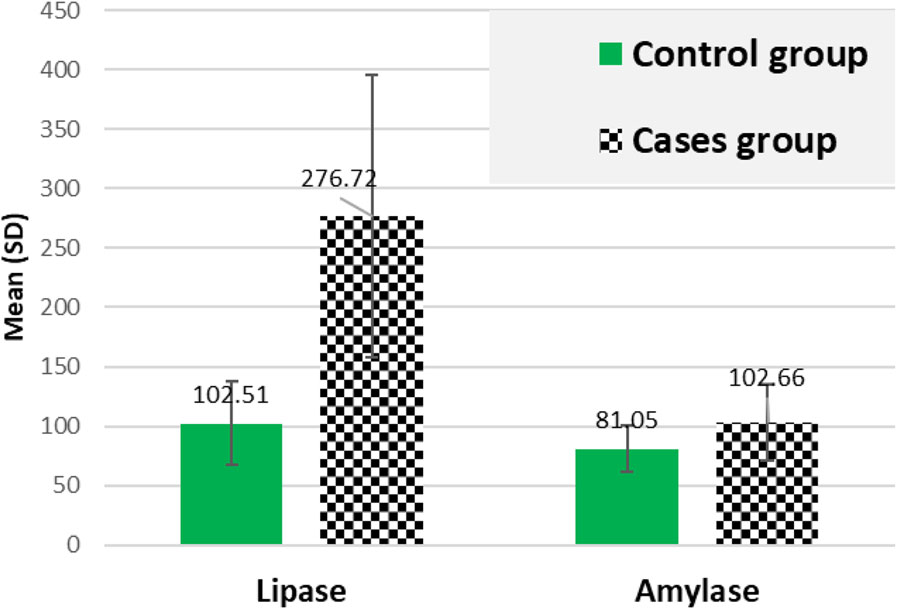
Significant differences in the pancreatic enzymes between the studied groups

Inflammatory markers exhibited significant differences between the study groups indicating persistent long-term inflammatory process residue after negative RT-PCR test of SARS-CoV-2. Table 2 shows significant higher values of ESR, CRP, ferritin and D-dimer (*P* < 0.001) in COVID-19 survivors than control peers. Moreover, lymphocytic count percentage was significantly reduced in the case group (*P* < 0.001), along with a significant elevation of neutrophil count percentage (*P* < 0.001). We noticed significant higher values of haemoglobin concentration in COVID-19 survivors (*P* =0.042). COVID-19 survivors exhibited significant higher values of ALT, AST, GGT (*P* < 0.001), and ALP (*P* = 0.001) but with statistically significant reduction of serum albumin (*P* < 0.001). Meanwhile, serum bilirubin revealed a non-significant difference between the study groups (*P* =0.556) (see Table 3 and Fig. 1) Exocrine pancreatic enzymes; serum lipase and amylase showed statistically significant elevations in COVID-19 survivors (*P* = 0.001 and *P* < 0.001), respectively as shown in Table 3 and Fig. 2. COVID-19 survivors experienced significantly elevated BUN (*P* =0.025), serum creatinine (*P* =0.025), and urine ACR (*P* =0.001) along with a significant reduction of eGFR (*P* =0.006). 25(OH) vitamin D was significantly reduced (*P* =0.001) but serum uric acid, triglycerides and LDL-cholesterol showed significant higher values (P =0.001) in COVID-19 survivors than control peers (see Table 3).

**Table 2:** Inflammatory and haematological characteristics of participants

Variable	Control *N* = 120	Cases *N* = 120	Test of significance	*P* value
	Mean ± SD			
ESR (mm/h)	11.84±2.96	44.78±20.99	*t*=12.35	*p*< 0.001*
CRP (mg/L) (Min-Max, IQR)	7.9(4.5–12.0) (7.0–9.5)	15.75(4.5–136.0) (10.70–28.5)	*z*=8.25	*p*=0.001*
D.dimer	0.336±0.161	0.494±0.257	*t*=4.35	*p*< 0.001*
Ferritin (ng/mL)	19.29±6.16	212.44±81.57	*t*=18.74	*p*< 0.001*
WBCS ( × 10^9^/L)	5.82±1.59	7.63±3.51	*t*=3.82	*p*< 0.001*
RBCS (million/mcL)	4.82±0.64	4.87±0.63	*t*=0.528	*p*=0.598
HB (gm/dL)	12.63±1.72	13.21±1.74	*t*=2.05	*p*=0.042*
HCT(%)	37.99±4.51	38.89±5.25	*t*=1.12	*p*=0.265
MCV (fL)	79.53±7.68	80.61±8.30	*t*=0.817	*p*=0.415
MCH (pg)	26.40±3.21	27.49±3.07	*t*=2.11	*p*=0.036*
MCHC (g/dL)	33.08±1.19	34.24±1.19	*t*=5.95	*p*=0.001*
RDW (%)	14.69±2.10	14.12±1.68	*t*=1.88	*p*=0.062
PLT (× 10^3^/ mcL)	238.32±75.23	289.24±131.47	*t*=2.77	*p*=0.006*
MPV (fL)	8.62±0.81	8.52±1.02	*t*=0.643	*p*=0.521
Neutrophil count percentage	50.13±11.61	74.37±17.48	*t*=9.62	*p*=0.001*
Lymphocyte count percentage	37.0±10.04	16.92±14.63	*t*=9.45	*p*=0.001*

*t*: Student’s *t* test, *Z*: Mann-Whitney *U* test, *statistically significant (if *p* < 0.05). All parameters described as mean ± SD, Median (min-max and IQR). *HCT* haematocrit %.

**Table 3:** Biochemical findings of the study groups

Variable	Control *N*=120	Cases *N*=120	Test of significance	*P* value
	Mean ± SD			
ALT (IU/L)	20.92±7.03	76.76±28.52	*t*=15.21	*p*< 0.001*
AST (IU/L)	18.62±5.8	57.02±28.81	*t*=10.42	*p*< 0.001*
GGT (IU/L)	32.19±10.16	88.77±29.76	*t*=14.49	*p*< 0.001*
Bilirubin (mg/dL)	0.965±0.186	0.987±0.244	*t*=0.590	*p*=0.556
Albumin(g/dL)	4.74±0.46	4.25±0.52	*t*=6.0	*p*=0.001*
ALP (IU/L)	57.06±13.29	88.70±28.41	*t*=8.22	*p*=0.001*
Lipase(U/L)	102.51±35.13	276.72±118.56	*t*=11.31	*p*=0.001*
Amylase(U/L)	81.05±19.83	102.66±31.99	*t*=4.76	*p*< 0.001*
BUN (mg/dL)	13.22±3.82	23.51±4.27	*t*=2.26	*p*=0.025*
Serum creatinine (mg/dL)	1.03±0.13	1.09±0.19	*t*=2.26	*p*=0.025*
eGFR (ml/ min/1.73m^2^)	84.46±14.18	75.80±21.48	*t*=2.80	*p*=0.006*
Urine ACR (mg/g)	12.69±4.23	23.57±8.44	*t*=9.42	*p*=0.001*
25(OH) Vitamin D3 (ng/mL)	40.32±11.76	23.22±8.45	*t*=10.47	*p*=0.001*
Calcium (mg/dL)	9.31±0.54	9.19±0.66	*t*=1.189	*p*=0.236
Magnesium (mg/dL)	1.95±0.20	2.13±0.22	*t*=5.23	*p*< 0.001*
Phosphorus (mg/dL)	3.45±0.60	4.44±0.60	*t*=10.01	p< 0.001*
Total cholesterol (mg/dL)	168.75±33.22	209.90±32.04	*t*=7.70	*p*=0.001*
Triglycerides (mg/dL)	132.21±43.48	214.54±62.21	*t*=9.07	*p*=0.001*
HDL-C (mg/dL)	45.78±13.14	42.98±8.98	*t*=1.57	*p*=0.119
LDL-C (mg/dL)	96.90±30.83	124.80±30.06	*t*=5.59	*p*=0.001*
VLDL-C (mg/dL)	26.90±10.69	42.50±12.21	*t*=8.18	*p*=0.001*
Uric acid (mg/dL)	4.48±0.98	6.32±1.06	*t*=10.96	*p*=0.001*`

*t*: Student’s *t* test *statistically significant (if *p* < 0.05). All parameters described as mean ± SD

Multivariate regression analysis with adjusted odds ratio (AOR: 95% CI) showed that ESR (AOR =2.06 , 95% CI= 1.158–3.67, *P* = 0.014), haemoglobin concentration ( AOR= 3.09, 95% CI =1.06–9.05, *P* = 0.039), serum lipase (AOR= 1.11, 95% CI =1.02–1.22, *P* = 0.018), BUN (AOR =2.47, 95% CI =1.35-4.52, P = 0.003), urine ACR (AOR= 1.159, 95% CI =1.003–1.34, *P* = 0.046), 25(OH) vitamin D (AOR =0.789, 95% CI =0.679–0.917, *P* = 0.002), and serum uric acid (AOR= 12.23, 95% CI =2.17–68.99, *P* = 0.005) were the significant predictors of COVID-19 survivors (94.8% an overall prediction) as shown in Table 4.

**Table 4:** Binary logistic regression in prediction of COVID-19 survivors

	Univariate analysis		Multivariate analysis	
	*p*	COR (95%CI)	*p*	AOR (95%CI)
SBP (mmHg)	< 0.001*	1.07 (1.03–1.109)	0.839	1.02 (0.816–1.28)
ESR (mm/h)	< 0.001*	1.76 (1.39–2.22)	0.014*	2.06 (1.158–3.67)
CRP (mg/L)	< 0.001*	1.72 (1.39–2.11)	0.078	3.04 (0.882–10.47)
D.dimer	< 0.001*	76.66 (9.04–650.04)		
WBCS (× 10^9^/L)	0.001*	1.28 (1.11–1.47)	0.06	2.33 (0.97–5.65)
HB (gm/dL)	0.04*	1.21 (1.0–1.47)	0.039*	3.09 (1.06–9.05)
MCH (pg)	0.04*	1.12 (1.01–1.24)	0.297	1.47 (0.709–3.09)
MCHC (g/dL)	< 0.001*	2.29 (1.64–3.21)	0.643	1.47 (0.709–3.08)
PLT (× 10^3^/ mcL)	0.009*	1.005 (1.001–1.008)	0.486	0.977 (0.915–1.04)
Neutrophils %	< 0.001*	1.09 (1.06–1.125)	0.338	1.22 (0.813–1.83)
Lymphocytes %	< 0.001*	0.901 (0.873–0.931)	0.177	0.533 (0.213–1.33)
Magnesium (mg/dL)	< 0.001*	58.39 (10.0–340.93)	0.351	106.72 (undefined)
Phosphorus (mg/dL)	< 0.001*	12.00 (5.49–26.20)	0.246	5.69 (0.301–107.68)
ALT (IU/L)	0.002*	1.57 (1.172–2.1)	0.095	1.48 (0.934–2.33)
AST (IU/L)	< 0.001*	1.29 (1.175–1.414)	0.336	1.185 (0.839–1.67)
GGT (IU/L)	< 0.001*	1.175 (1.10–1.25)	0.623	1.04 (0.89–1.21)
Albumin (g/dL)	< 0.001*	0.153 (0.073–0.317)	0.744	1.55 (0.112–21.55)
ALP (IU/L)	< 0.001*	1.07 (1.042–1.09)	0.236	0.912 (0.782–1.06)
Lipase (U/L)	< 0.001*	1.025 (1.016–1.034)	0.018*	1.11 (1.02–1.22)
Amylase (U/L)	< 0.001*	1.03 (1.015–1.043)	0.051	0.844 (0.712–1.0)
BUN (mg/dL)	< 0.001*	1.96 (1.56–2.47)	0.003*	2.47 (1.35–4.52)
Creatinine (mg/dL)	0.028*	9.68 (1.27–73.56)	0.136	Undefined
eGFR	0.008*	0.975 (0.957–0.994)	0.187	0.904 (0.777–1.05)
Urine ACR (mg/g)	< 0.001*	1.33 (1.21–1.46)	0.046*	1.159 (1.003–1.34)
25(OH) VIT D (ng/mL)	< 0.001*	0.822 (0.773–0.875)	0.002*	0.789 (0.679–0.917)
TC (mg/dL)	< 0.001*	1.04 (1.03–1.05)	0.984	1.00 (0.902–1.11)
TG (mg/dL)	< 0.001*	1.03 (1.02–1.04)	0.122	1.047 (0.988–1.11)
LDL (mg/dL)	< 0.001*	1.03 (1.02–1.05)	0.256	1.08 (0.946–1.23)
VLDL (mg/dL)	< 0.001*	1.147 (1.09–1.203)	0.726	0.957 (0.746–1.23)
Uric acid (mg/dL)	< 0.001*	5.56 (3.29–9.39)	0.005*	12.23 (2.17–68.99)

Overall % predicted=94.8%, model *χ*^2^=26.19, *significant value, *COR* crude odds ratio, *AOR* adjusted odds ratio, *95%CI* 95% confidence interval

## Discussion

Pattern of liver injury during acute COVID-19 had been addressed enormously, meanwhile, the long-term impact of COVID-19 on the hepatic functions is still unclear. In the current study, Liver function tests showed statistically significant variations in COVID-19 survivors than individuals without history of COVID-19 exposure. ALT, AST, GGT, and ALP were significantly elevated in COVID-19 survivors. On the other hand, although normal values of serum albumin, we noticed statistically significant reduction of serum albumin in COVID-19 survivors. Interestingly, serum bilirubin showed non-significant difference between the study groups.

Ya-Wen et al. [10] found elevated levels of ALT, GGT and ALP along with reduced serum albumin in COVID-19 survivors for a period of 14 days after discharge but with gradual normalization of these parameters within two months. In our study, we found a persistent elevation of ALT, AST, GGT, and ALP for 3 month after resolved COVID-19. The former study reported dropout of the majority of COVID-19 survivors during the follow-up period.

Fan et al. [11] studied disruption of liver function in COVID-19 patients and reported reversible mild to moderate elevation of hepatic aminotransferases without concomitant elevation of serum total bilirubin. Also, Xu et al. [12] reported a non-elevated serum bilirubin in COVID-19 patients, despite the abundant expression of ACE2 in the hepatic vascular endothelium, cholangiocytes rather than the hepatocytes. They attributed these findings to the overwhelming systemic inflammatory response rather than direct viral invasion. However, the precise pathogenesis of liver injury in COVID-19 patients is still controversial. Multiple theories were postulated to explain the pathogenesis of hepatic changes such as ACE2-mediated direct hepatocyte viral invasion, disrupted immune homeostasis, systemic inflammatory response, concurrent hypotension, pneumonia-associated hypoxia, cytokine storm with a surge of the pro-inflammatory cytokines, and drug-induced hepatotoxicity [13, 14]. In addition, Xu et al. [15] reported moderate micro-vesicular steatosis, lobular and portal tract inflammatory infiltrates with significant reduction of CD4 and CD8 cells in liver biopsies of COVID-19 patients. Also, Tian et al. [16] found mild sinusoidal dilatation, focal macro-vesicular steatosis without reliable evidence of bile duct damage. Moreover, Zsuzsanna et al. [17] attributed these alterations to the lymphocytic endothelitis with hepatocyte necrosis induced by direct vial invasion and immune cell hyper-activation.

In our study, COVID-19 survivors experienced mild but significant elevated serum lipase and amylase than healthy counterparts. This finding may be explained by the copious expression of ACE2 receptors within the pancreatic tissue. In agreement with Furong et al. [18] and Fan et al. [19] who considered SARS-COV-2 as a potential viral cause of pancreatitis through direct cytopathic effect as well as the systemic inflammatory response, disrupted immune system with cytokine surge, and potential drug-induced pancreatitis.

In our study, BUN, serum creatinine, and urine albumin creatinine ratio were significantly elevated along with relatively reduced eGFR in COVID-19 survivors than others without a history of COVID-19. In accordance with Xu-Wei et al*.* [20] who carried out a case-series study for 12 patients with COVID-19 and reported significantly elevated serum creatinine, BUN, and micro-albuminuria during COVID-19 illness with partial improvement over a period more than 1 month after recovery. These findings were explained by Zou X et al. [21] who reported high expression of ACE2 in the apical membrane of the epithelial cells at the proximal renal tubules.

We found significantly reduced lymphocyte count percentage in COVID-19 survivors than non-COVID-19 exposed peers. Lowered lymphocytic count is a common association and sequence of various coronavirus infection. Wong et al. [22] reported reduced lymphocyte count in the majority of patients with SARS-CoV. Furthermore, the lymphocytic count was inversely correlated with severity of COVID-19 [23]. The direct invasion and lysis of lymphocytes by SARS-CoV-2 via ACE2 receptors located on their surface, enhanced lymphocyte apoptosis triggered by the systemic inflammatory response with pro-inflammatory cytokines surge and lymphoid organs atrophy were hypothesized to explain the lowered lymphocytic count during and after COVID-19 [24].

On the other hand, Holshue et al. [25] studied the first case of COVID-19 in the United States and reported rapid reversal of reduced lymphocytic count after 2 weeks of acute illness. On contrary, COVID-19 survivors in the current study showed persistently lowered lymphocytic count for more than 3 months after recovery.

Our results revealed persistent higher levels of inflammatory markers such as ESR, CRP, D-dimer, and ferritin in COVID-19 survivors denoting residual systemic inflammatory response. Despite different study design, our results are consistent with Sandra et al. [26] who conducted meta-analysis of the long-term effects of COVID-19. They found residual elevation of CRP, D-dimer, and ferritin associating post-COVID-19 related symptoms despite the heterogeneity of their data.

Our results agreed with Mandal et al. [27] who carried out a follow-up observational study for COVID-19 survivors during a period of 4 to 6 weeks after discharge. They found persistent elevated inflammatory markers such as D-dimer and CRP. Also, Sonnweber et al. [28] found persistent high ferritin levels among COVID-19 survivors 2 months after the onset of COVID-19.

In the current study, systolic blood pressure was significantly elevated in COVID-19 survivors. COVID-19 pandemic may increase fear, anxiety, socio-economic burdens, mental disorders, and decrease physical activity. Therefore, it can potentially compromise blood pressure control [29].

We strived to detect the pathological clinical and biochemical residue after COVID-19 recovery in our locality. Our strength points were the relatively longer follow-up period after the negative RT-PCR test of SARS-CoV-2. Strictly, the case group included COVID-19 survivors with a history of mild to moderate illness. We tried to avoid confounders such as severe COVID-19 illness as well as a history of acute or chronic morbidities after recovery till enrolment time. We faced many limitations such as the lack of documented data of COVID-19 survivors before and during the acute stage of illness, we relied on the detailed medical history of participants. However honestly, we cannot guarantee full trust in participants’ story. Moreover, the scarcity of similar trials due to novelty of SARS-CoV-2 and the single-centre study represented major limitations. In this study, we tried to detect the significant differences between COVID-19 survivors who are expected to be definitely healthy after pre-specified convalescence period versus healthy subjects without COVID-19 exposure. Multi-centre trials with larger-scale, multiple ethnicities, longer follow-up period and invasive tools may help more advanced research in the future.

## Conclusion

COVID-19 survivors experienced residual significant clinical and biochemical alterations that necessitate comprehensive medical follow-up care for longer periods.

## Data Availability

The datasets used and/or analysed during the current study are available from the corresponding author on reasonable request.

## Abbreviations

COVID-19: Coronavirus disease 19
SARS-CoV-2: Severe acute respiratory syndrome coronavirus 2
SARS-CoV: Severe acute respiratory syndrome coronavirus
MERS-CoV: Middle East respiratory syndrome coronavirus
RT-PCR: Real-time reverse transcriptase-polymerase chain reaction
ALT: Alanine aminotransferase
AST: Aspartate aminotransferase
GGT: Gamma-glutamyl transpeptidase
urine ACR: Urine albumin/creatinine ratio
ACE2: Angiotensin-converting enzyme 2
